# Supplementary material for: Deep Learning Models for Predicting Gas Adsorption Capacity of Nanomaterials
Source: Nanomaterials (Basel). 2022 Sep 27;12(19):3376. doi: 10.3390/nano12193376 (PMC9565823; doi:10.3390/nano12193376)
Supplement: Supplementary file 1 [file nanomaterials-12-03376-s001.zip › nanomaterials-1868340-supplementary.pdf]

nanomaterials

Article

Deep Learning Models for Predicting Gas Adsorption Capacity of Nanomaterials

Table S1: Hyperparameter tuning in long short-term memory (LSTM)
Table S2: Hyperparameter tuning in multilayer perceptron (MLP)
Table S3: Holdout validations performance of models with and without accessible pore volumes for predicting methane adsorption at 1 bar 6
Figure S1:Workflow of developing deep learning models
Figure S2: Workflow of 10 iterations of 10-fold cross validation
Figure S3: Workflow of 100 iterations of holdout validation
Figure S4: Performance of MLP model in 10-fold validations for methane adsorption at 65 bar
Figure S5: Performance of LSTM model in 10-fold validations for methane adsorption at 65 bar
Figure S6: Performance of MLP model in 10-fold validations for methane adsorption at 35 bar
Figure S7: Performance of LSTM model in 10-fold validations for methane adsorption at 35 bar
Figure S8: Performance of MLP model in 10-fold validations for methane adsorption at 5.8 bar
Figure S9: Performance of LSTM model in 10-fold validations for methane adsorption at 5.8 bar
Figure S10: Performance of MLP model in 10-fold validations for methane adsorption at 1 bar
Figure S11: Performance of LSTM model in 10-fold validations for methane adsorption at 1 bar
Figure S12: Performance of MLP model in 100 repeated holdout validations for methane adsorption at 65 bar
Figure S13: Performance of LSTM model in 100 repeated holdout validations for methane adsorption at 65 bar
Figure S14: Performance of MLP model in 100 repeated holdout validations for methane adsorption at 35 bar
Figure S15: Performance of LSTM model in 100 repeated holdout validations for methane adsorption at 35 bar
Figure S16: Performance of MLP model in 100 repeated holdout validations for methane adsorption at 5.8 bar
Figure S17: Performance of LSTM model in 100 repeated holdout validations for methane adsorption at 5.8 bar
Figure S18: Performance of MLP model in 100 repeated holdout validations for methane adsorption at 1 bar
Figure S19: Performance of LSTM model in 100 repeated holdout validations for methane adsorption at 1 bar
Figure S20: Performance of LSTM model using 26 descriptors in 100 repeated holdout validations for methane adsorption at 1 bar
Figure S21: Performance of MLP model using 26 descriptors in 100 repeated holdout validations for methane adsorption at 1 bar
Figure S22: Performance of MLP model in 10-fold cross validations for carbon dioxide adsorption at 2.5 bar
Figure S23: Performance of LSTM model in 10-fold cross validations for carbon dioxide adsorption at 2.5 bar

Figure S24: Performance of MLP model in 10-fold cross validations for carbon dioxide adsorption at 0.5 bar
Figure S25: Performance of LSTM model in 10-fold cross validations for carbon dioxide adsorption at 0.5 bar
Figure S26: Performance of MLP model in 10-fold cross validations for carbon dioxide adsorption at 0.05 bar
Figure S27: Performance of LSTM model in 10-fold cross validations for carbon dioxide adsorption at 0.05 bar
Figure S28: Performance of MLP model in 100 repeated holdout validations for carbon dioxide adsorption at 2.5 bar
Figure S29: Performance of LSTM model in 100 repeated holdout validations for carbon dioxide adsorption at 2.5 bar
Figure S30: Performance of MLP model in 100 repeated holdout validations for carbon dioxide adsorption at 0.5 bar
Figure S31: Performance of LSTM model in 100 repeated holdout validations for carbon dioxide adsorption at 0.5 bar
Figure S32: Performance of MLP model in 100 repeated holdout validations for carbon dioxide adsorption at 0.05 bar
Figure S33: Performance of LSTM model in 100 repeated holdout validations for carbon dioxide adsorption at 0.05 bar
Figure S34: Performance of MLP model in 100 repeated holdout validations for methane adsorption in the mixture of MOFs and COFs at 65 bar
Figure S35: Performance of LSTM model in 100 repeated holdout validations for methane adsorption in the mixture of MOFs and COFs at 65 bar

Table S1. Hyperparameter tunning in long short-term memory (LSTM).

Number of neurons	Number of hidden Layers	Batch Size	r^2	Time (h)
200	1	16	0.966	7.2
100	1	16	0.966	3.5
300	1	16	0.967	17.5
50	1	16	0.964	2.7
100	2	16	0.970	6.6
100	3	16	0.968	9.5
100	2	8	0.970	11.8
100	2	32	0.970	4.6
100	2	48	0.969	4.5

r^2: correlation coefficient. Three hyperparameters (number of neurons, number of layers and batch size) were tuned using 10 repeated 5-fold cross validation for methane adsorption in a mixture dataset containing 5000 MOFs and 5000 COFs. The set of three hyperparameters that produced the highest correlation coefficient r^2 was selected to construct our final model. First, we isolated the number of neurons and search for the best possible value while keeping other hyperparameters fixed (number of LSTM layers = 1, batch size = 16). We started with 200 neurons and the coefficient of determination (r^2) was at 0.966 and the time needed for this process was 7 hours. Next, we increased the number of neurons to 300 and found r^2 at 0.967, and this process required 17 hours. We decreased the number of neurons to 100 and found r^2 was around 0.966 and the running time dropped to 3 hours. We further decreased the number of neurons to 50, and r^2 was found to be at 0.964. Considering the time cost and the accuracy of the models, we picked 100 neurons as our optimal number. Next, we searched for the best-performing models with a varied number of hidden LSTM layers with 100 neurons in each layer and batch size of 16. Since we already evaluated the model with 1 hidden layer, we increased the number of hidden layers to 2 and 3. The results showed that the model with 2 hidden LSTM layers had better accurate results with r^2 at 0.970. In the next two steps, we searched for the optimal batch size, we started with 16 and found batch size 32 gave the best results. Therefore, we selected LSTM models with 100 neurons, 2 hidden layers, and batch sizes of 32 to construct our final model (highlighted in the table).

Table S2. Hyperparameter tunning in multilayer perceptron (MLP).

Number of neurons	Number of hidden layers	alpha	r^2	Time (h)
20	3	0.3	0.961	0.2
500	3	0.3	0.971	10.5
300	3	0.3	0.970	2.5
300	10	0.3	0.967	7.0
300	1	0.3	0.964	1.0
300	6	0.3	0.969	5.8
300	3	0.00001	0.970	1.3
300	3	1	0.970	1.7

r^2: correlation coefficient. We used 10-times-repeated 5-fold cross validation to tune three hyperparameters in the MLP model: number of neurons, number of layers, and alpha using methane adsorption in a mixture dataset containing 5000 MOFs and 5000 COFs. The set of parameters that produced the highest correlation coefficient (r^2) was used in the final model. First, we searched for the best possible values for the number of neurons while keeping the number of hidden layers at 3, alpha at 0.3. We started with 20 neurons and r^2 obtained was 0.961 and the time needed for this process was 0.2 hours. Next, we increased the number of neurons to 500 and found that r2 increased a little bit to 0.971 and took 10.5 hours. Then, we decreased the number of neurons to 300 and found r^2 was around 0.970 and the running time dropped to 2.5 hours. Considering the time cost and the accuracy of the models, we picked 300 neurons as our optimal number. Then we searched the number of layers while keeping the number of neurons at 300 and alpha at 0.3. The model with 3 hidden layers was found to have the maximal performance. Then we searched for alpha while keeping the number of layers at 3 and the number of neurons at 300. Since little variation was observed when changing alpha values, we maintained alpha values at 0.3 in our final model. The optimal combination of hyperparameters was found to be 3 hidden layers, 300 neurons, and alpha at 0.3 (highlighted in the table).

Table S3. Holdout validations performance of models with and without accessible pore volumes for predicting methane adsorption at 1 bar.

Model	Number of descriptors *	R^2	r^2	sMAE	sRMSE
LSTM	26	0.8649 ± 0.0064	0.8866 ± 0.0031	0.1492 ± 0.0014	0.3361 ± 0.0055
LSTM	25	0.8722 ± 0.0066	0.8902 ± 0.0030	0.1464 ± 0.0013	0.3306 ± 0.0052
LSTM	6	0.6148 ± 0.0239	0.7406 ± 0.0034	0.2894 ± 0.0018	0.5109 ± 0.0053
MLP	26	0.8800 ± 0.0071	0.8919 ± 0.0030	0.1550 ± 0.0056	0.3293 ± 0.0055
MLP	25	0.8755 ± 0.0120	0.8891 ± 0.0063	0.1526 ± 0.0063	0.3350 ± 0.0103
MLP	6	0.6951 ± 0.0335	0.7748 ± 0.0056	0.2760 ± 0.0112	0.4773 ± 0.0089

* - 5 descriptors: the dominant pore size, the max pore size, the void fraction, the gravimetric surface area, density; 6 descriptors: the 5 descriptors plus accessible volume; 25 descriptors: the 5 descriptors plus the 20 atom types; 26 descriptors: the 6 descriptors plus the 20 atom types..

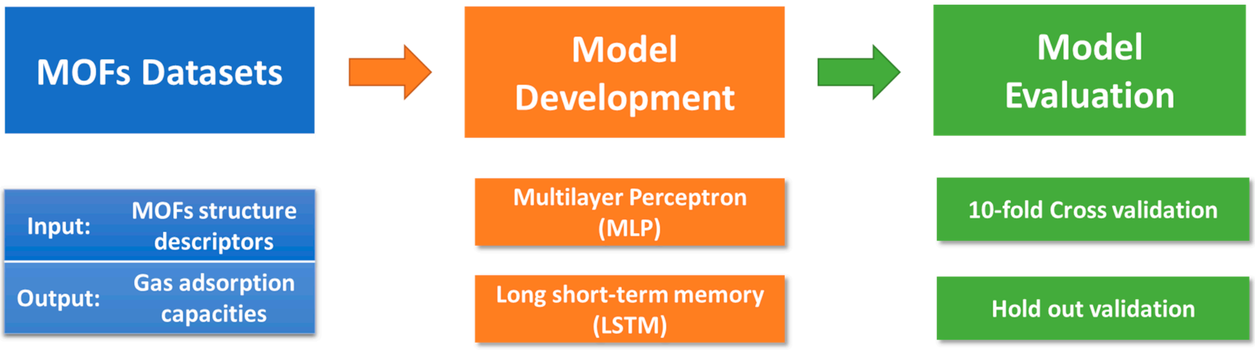

Figure S1. Workflow of developing deep learning models. The MOFs datasets were used to develop two deep learning models: multilayer perceptron (MLP) and long short-term memory (LSTM). The developed deep learning models were evaluated using two validation methods: 10-fold cross validation and holdout validation.

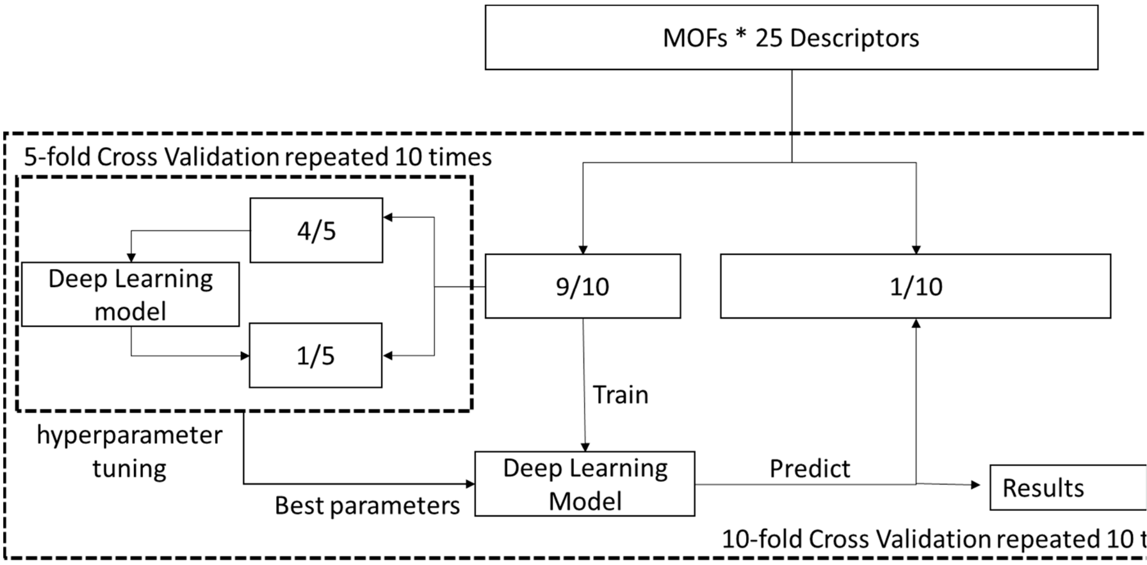

Figure S2. Workflow of 10 iterations of 10-fold cross validation. In 10-fold cross validation, the MOFs dataset was randomly divided into 10 groups. Nine groups were used to build deep learning (DL) models and the remaining group in the 10 groups was used to evaluate the constructed models. In the training process, 10 iterations of 5-fold cross validation were used to tune the hyperparameters used in the deep learning algorithms. The set of model hyperparameters that resulted in the best average performance was selected as the hyperparameters in the final model. This process was iterated 10 times so that each of the 10 groups was used as the test set once and only once. Then, the 10-fold cross validation was repeated 10 times to reach a statistically robust estimation of the model.

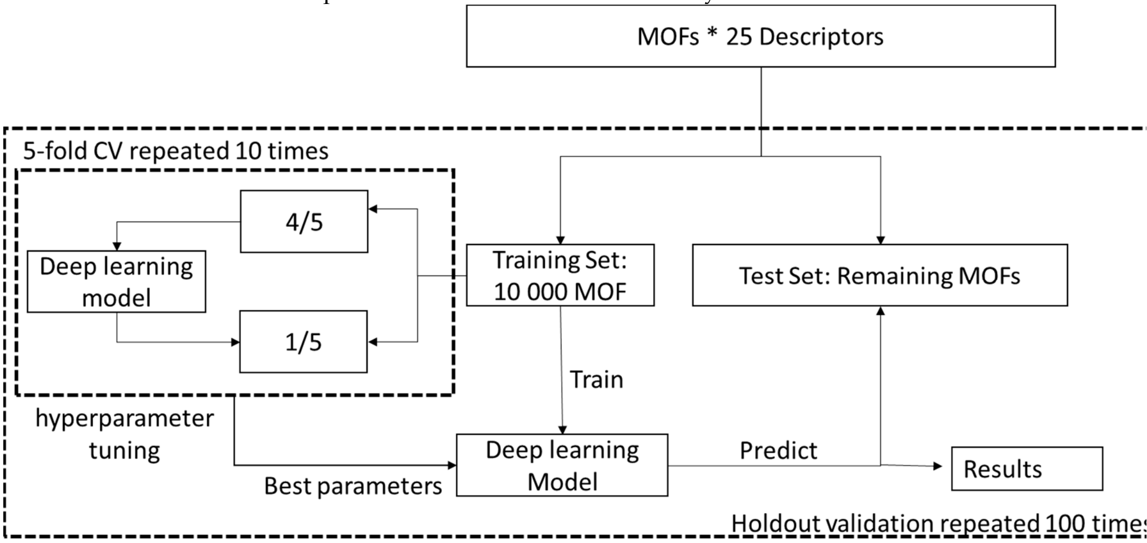

Figure S3. Workflow of 100 iterations of holdout validation. In the holdout validation, the MOFs dataset was randomly split into two sets: a training set with 10,000 MOFs and a test set containing the rest MOFs. The training set was used to train deep learning models and the test set was then used to evaluate the performance of the models. In the training process, 10 iterations of 5-fold cross validation were used to tune the hyperparameters used in the deep learning algorithms. The set of model hyperparameters that resulted in the best average performance was selected as the hyperparameters in the final model. The holdout validation was repeated 100 times to reach a statistically robust estimation of the model.

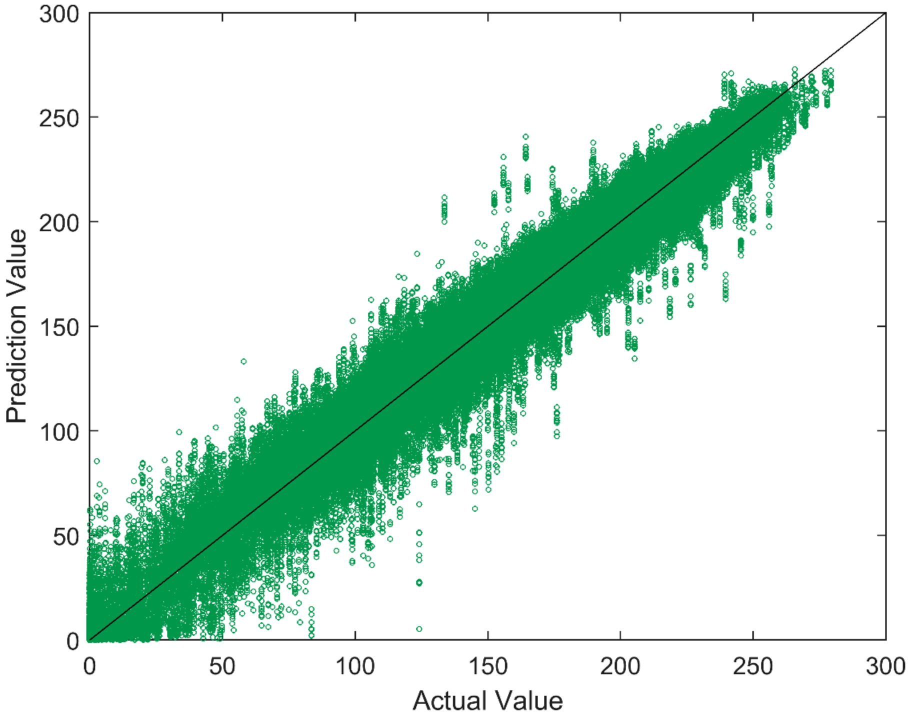

Figure S4. Performance of MLP model in 10-fold validations for methane adsorption at 65 bar. The x-axis gives the actual values of volumetric-based methane adsorption in MOFs in the unit of cm^3(STP)/cm^3 at 65 bar and the y-axis gives the predicted values from the MLP model. The prediction values were obtained from 10 repeated 10-fold cross validations. If a data point is on the diagonal line, the prediction and actual values are the same. .

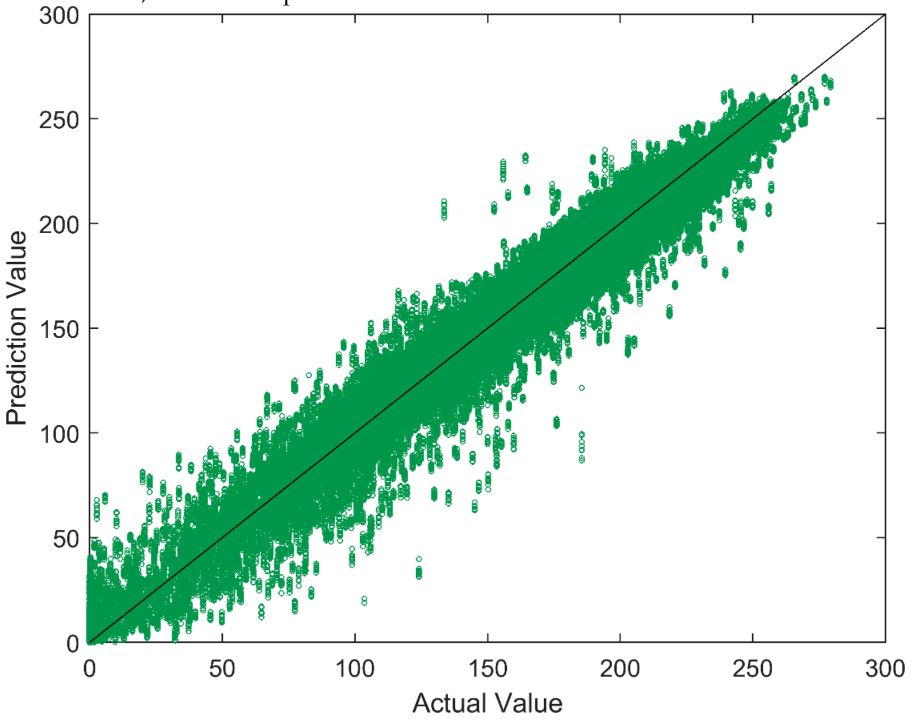

Figure S5. Performance of LSTM model in 10-fold validations for methane adsorption at 65 bar. The x-axis gives the actual values of volumetric-based methane adsorption in MOFs in units of cm^3(STP)/cm^3 at 65 bar and the y-axis gives the predicted values from the LSTM model. The prediction values were obtained from 10 repeated 10-fold cross validations. If a data point is on the diagonal line, the prediction and actual values are the same.

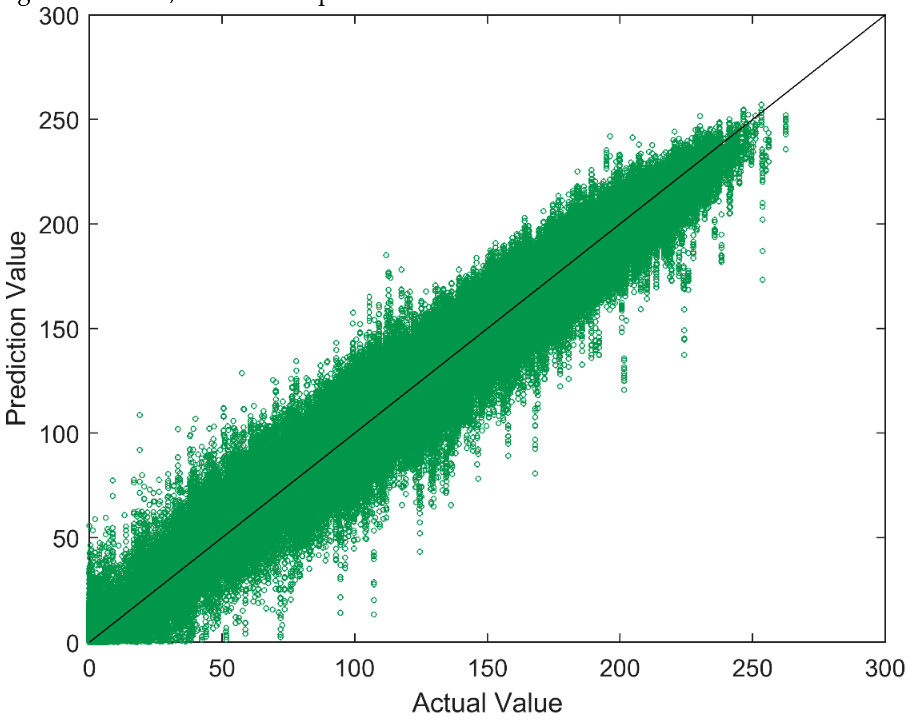

Figure S6. Performance of MLP model in 10-fold validations for methane adsorption at 35 bar. The x-axis gives the actual values of volumetric-based methane adsorption in MOFs in units of cm^3(STP)/cm^3 at 35 bar and the y-axis gives the predicted values from the MLP model. The prediction values were obtained from 10 repeated 10-fold cross validations. If a data point is on the diagonal line, the prediction and actual values are the same.

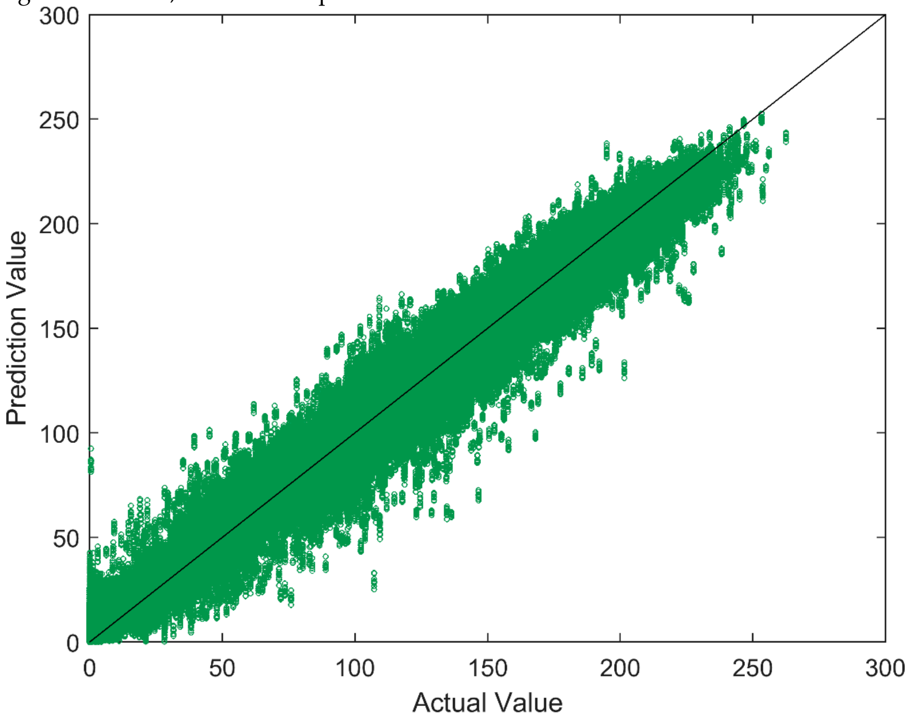

Figure S7. Performance of LSTM model in 10-fold validations for methane adsorption at 35 bar. The x-axis gives the actual values of volumetric-based methane adsorption in MOFs in units of cm^3(STP)/cm^3 at 35 bar and the y-axis gives the predicted values from the LSTM model. The prediction values were obtained from 10 repeated 10-fold cross validations. If a data point is on the

diagonal line, the prediction and actual values are the same.

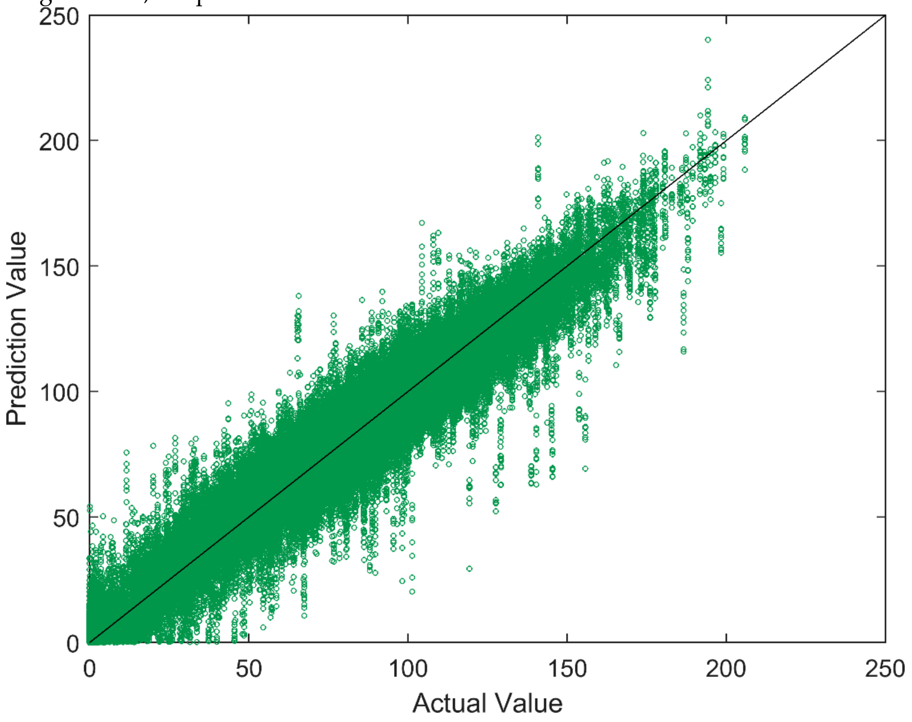

Figure S8. Performance of MLP model in 10-fold validations for methane adsorption at 5.8 bar. The x-axis gives the actual values of volumetric-based methane adsorption in MOFs in units of cm^3(STP)/cm^3 at 5.8 bar and the y-axis gives the predicted values from the MLP model. The prediction values were obtained from 10 repeated 10-fold cross validations. If a data point is on the diagonal line, the prediction and actual values are the same.

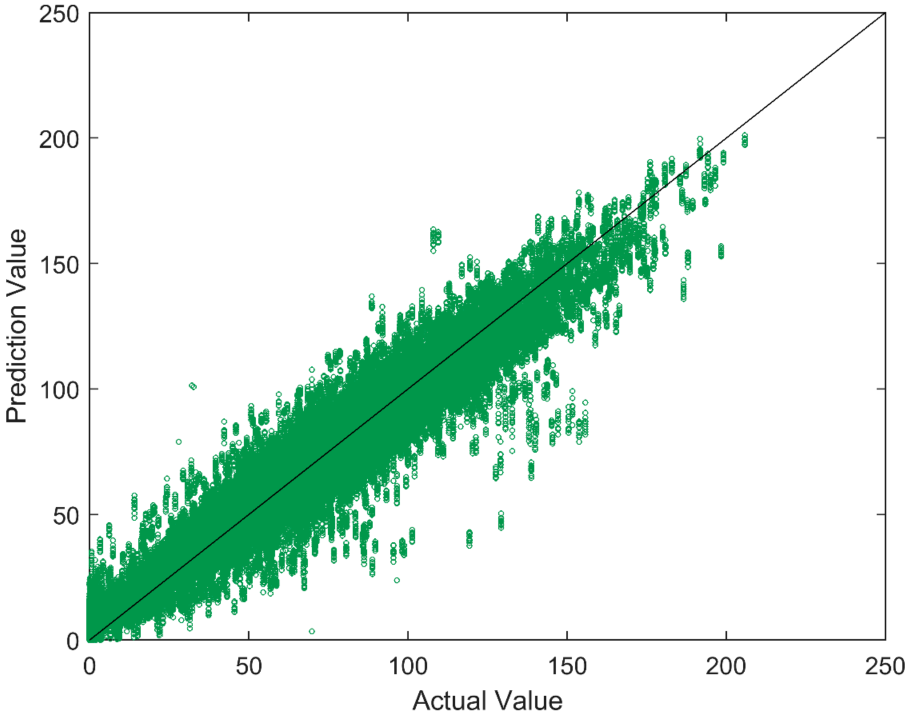

Figure S9. Performance of LSTM model in 10-fold validations for methane adsorption at 5.8 bar. The x-axis gives the actual values of volumetric-based methane adsorption in MOFs in units of cm^3(STP)/cm^3 at 5.8 bar and the y-axis gives the predicted values from the LSTM model. The prediction values were obtained from 10 repeated 10-fold cross validations. If a data point is on the diagonal line, the prediction and actual values are the same.

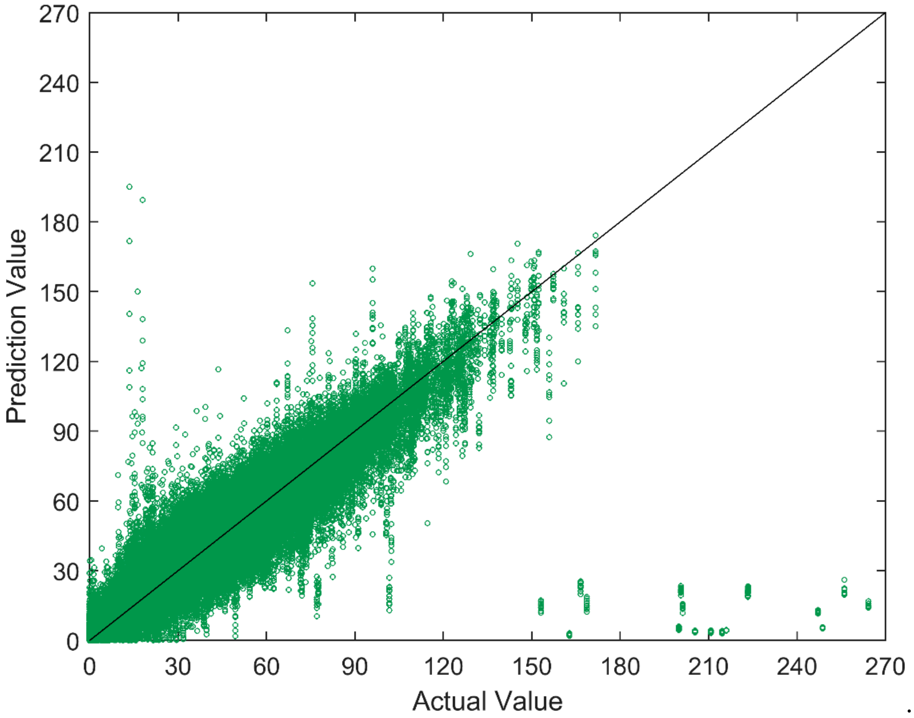

Figure S10. Performance of MLP model in 10-fold validations for methane adsorption at 1 bar. The x-axis gives the actual values of volumetric-based methane adsorption in MOFs in units of $cm^3(STP)/cm^3$ at 1 bar and the y-axis gives the predicted values from the MLP model. The prediction values were obtained from 10 repeated 10-fold cross validations. If a data point is on the diagonal line, the prediction and actual values are the same.

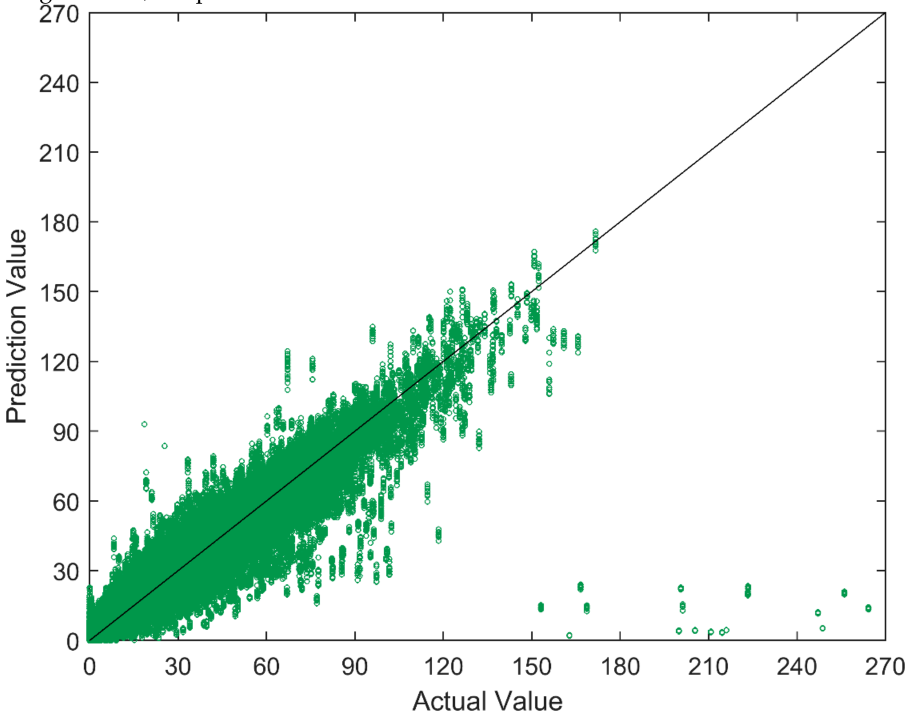

Figure S11. Performance of LSTM model in 10-fold validations for methane adsorption at 1 bar. The x-axis gives the actual values of volumetric-based methane adsorption in MOFs in units of $cm^3(STP)/cm^3$ at 1 bar and the y-axis gives the predicted values from the LSTM model. The prediction values were obtained from 10 repeated 10-fold cross validations. If a data point is on the

diagonal line, the prediction and actual values are the same.

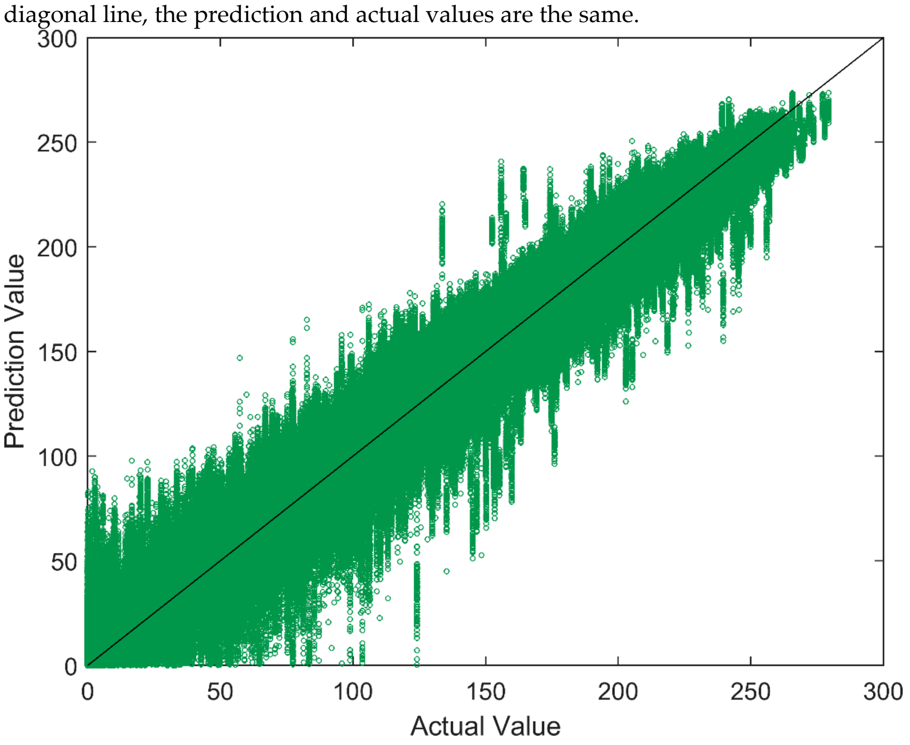

Figure S12. Performance of MLP model in 100 repeated holdout validations for methane adsorption at 65 bar. The x-axis gives the actual values of volumetric-based methane adsorption in MOFs in units of cm^3(STP)/cm^3 at 65 bar and the y-axis gives the predicted values from the MLP model. The prediction values were obtained from 100 repeated holdout validations. In the holdout validation, 10,000 MOFs were randomly selected as the training dataset to build the MLP model and the prediction values of the remaining MOFs were plotted in the figure. This procedure was repeated 100 times. If a data point is on the diagonal line, the prediction and actual values are the same.

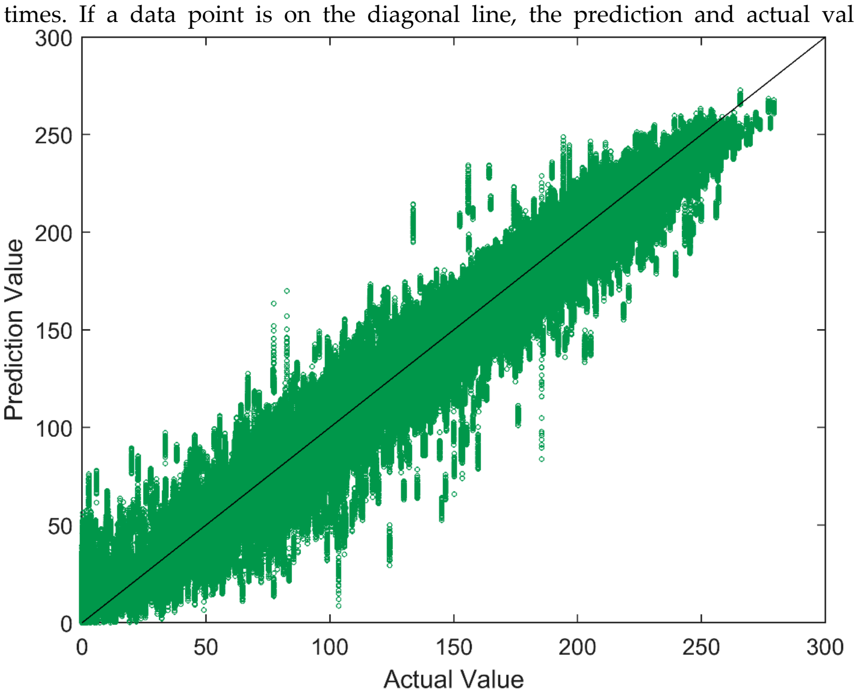

Figure S13. Performance of LSTM model in 100 repeated holdout validations for methane adsorption at 65 bar. The x-axis gives the actual values of volumetric-based methane adsorption in MOFs in units of cm^3(STP)/cm^3 at 65 bar and the y-axis gives the predicted values from the LSTM model. The prediction values were obtained from 100 repeated holdout validations. In the holdout validation, 10,000 MOFs were randomly selected as the training dataset to build the LSTM model and the prediction values of the remaining MOFs were plotted in the figure. This procedure was

repeated 100 times. If a data point is on the diagonal line, the prediction and actual values are the

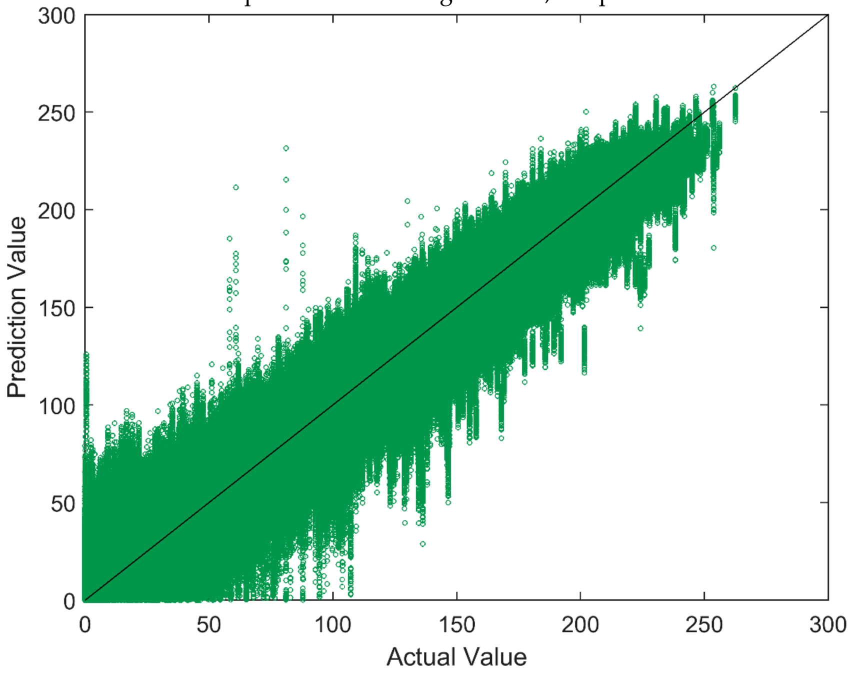

same.

Figure S14. Performance of MLP model in 100 repeated holdout validations for methane adsorption at 35 bar. The x-axis gives the actual values of volumetric-based methane adsorption in MOFs in units of cm^3(STP)/cm^3 at 35 bar and the y-axis gives the predicted values from the MLP model. The prediction values were obtained from 100 repeated holdout validations. In the holdout validation, 10,000 MOFs were randomly selected as the training dataset to build the MLP model and the prediction values of the remaining MOFs were plotted in the figure. This procedure was repeated 100 times. If a data point is on the diagonal line, the prediction and actual values are the same.

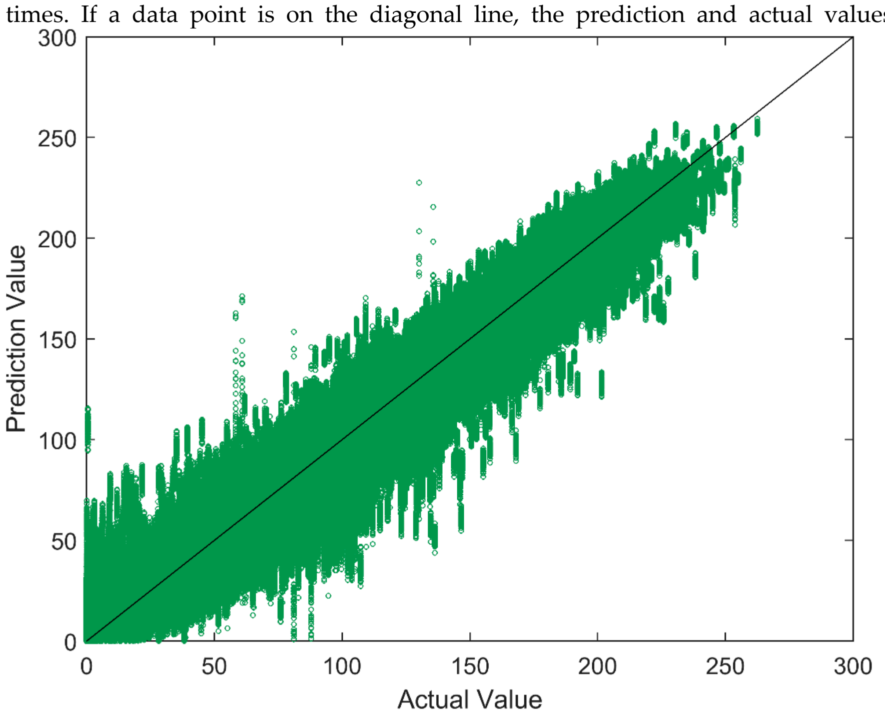

Figure S15. Performance of LSTM model in 100 repeated holdout validations for methane adsorption at 35 bar. The x-axis gives the actual values of volumetric-based methane adsorption in MOFs in units of cm^3(STP)/cm^3 at 35 bar and the y-axis gives the predicted values from the LSTM model. The prediction values were obtained from 100 repeated holdout validations. In the holdout validation, 10,000 MOFs were randomly selected as the training dataset to build the LSTM model and the prediction values of the remaining MOFs were plotted in the figure. This procedure was repeated

100 times. If a data point is on the diagonal line, the prediction and actual values are the same.

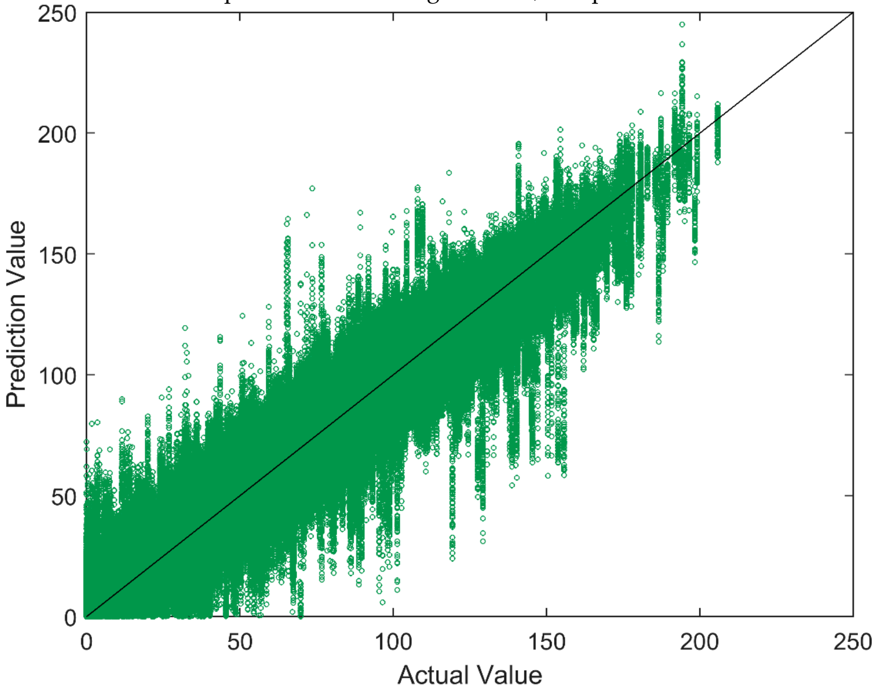

Figure S16. Performance of MLP model in 100 repeated holdout validations for methane adsorption at 5.8 bar. The x-axis gives the actual values of volumetric-based methane adsorption in MOFs in units of cm^3(STP)/cm^3 at 5.8 bar and the y-axis gives the predicted values from the MLP model. The prediction values were obtained from 100 repeated holdout validations. In the holdout validation, 10,000 MOFs were randomly selected as the training dataset to build the MLP model and the prediction values of the remaining MOFs were plotted in the figure. This procedure was repeated 100 times. If a data point is on the diagonal line, the prediction and actual values are the same.

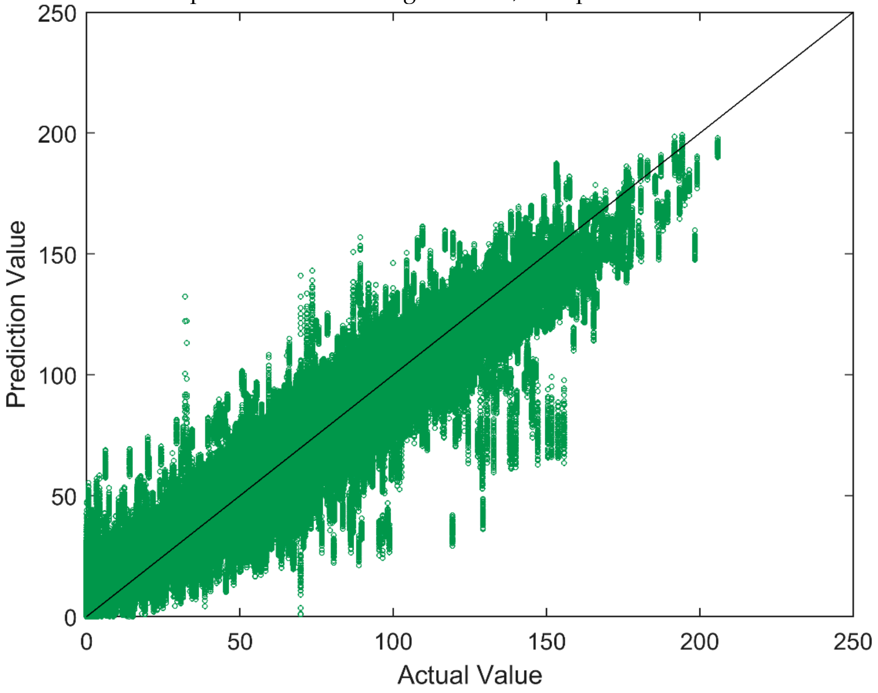

Figure S17. Performance of LSTM model in 100 repeated holdout validations for methane adsorption at 5.8 bar. The x-axis gives the actual values of volumetric-based methane adsorption in MOFs in units of cm^3(STP)/cm^3 at 5.8 bar and the y-axis gives the predicted values from the LSTM model. The prediction values were obtained from 100 repeated holdout validations. In the holdout validation, 10,000 MOFs were randomly selected as the training dataset to build the LSTM model and the prediction values of the remaining MOFs were plotted in the figure. This procedure was repeated

100 times. If a data point is on the diagonal line, the prediction and actual values are the same.

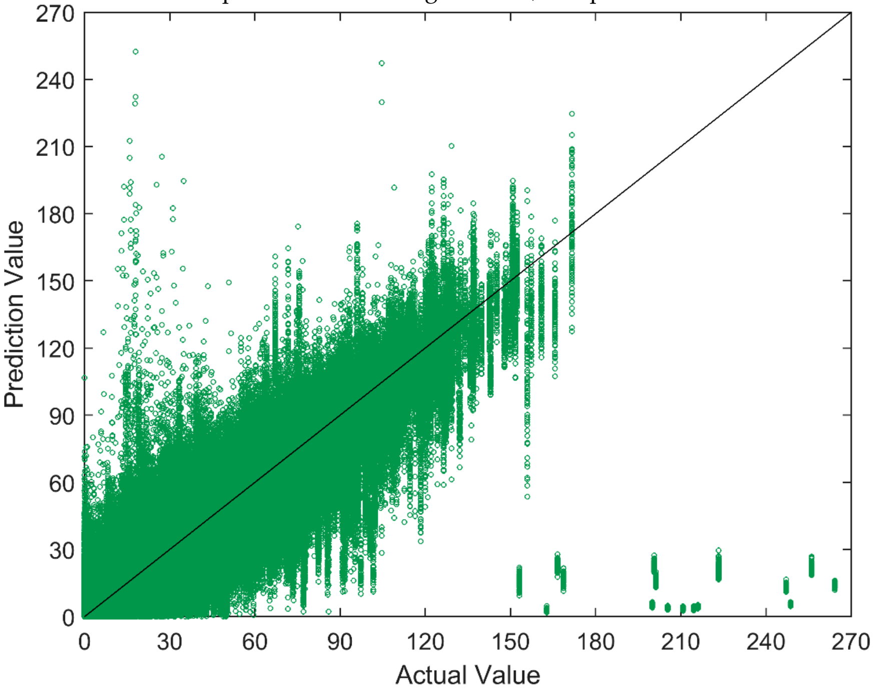

Figure S18. Performance of MLP model in 100 repeated holdout validations for methane adsorption at 1 bar. The x-axis gives the actual values of volumetric-based methane adsorption in MOFs in units of cm^3(STP)/cm^3 at 1 bar and the y-axis gives the predicted values from the MLP model. The prediction values were obtained from 100 repeated holdout validations. In the holdout validation, 10,000 MOFs were randomly selected as the training dataset to build the MLP model and the prediction values of the remaining MOFs were plotted in the figure. This procedure was repeated 100 times. If a data point is on the diagonal line, the prediction and actual values are the same.

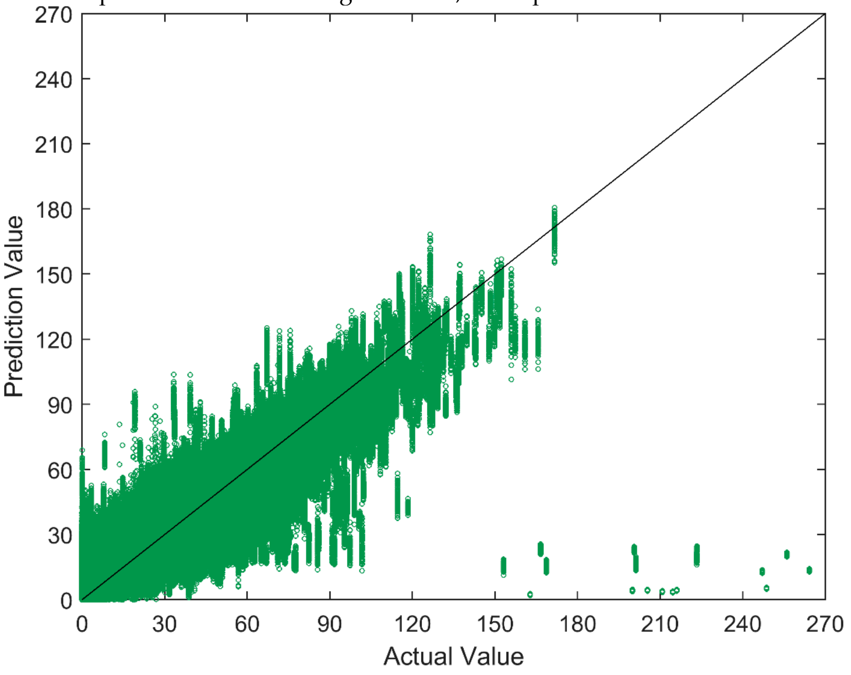

Figure S19. Performance of LSTM model in 100 repeated holdout validations for methane adsorption at 1 bar. The x-axis gives the actual values of volumetric-based methane adsorption in MOFs in units of cm^3(STP)/cm^3 at 1 bar and the y-axis gives the predicted values from the LSTM model. The prediction values were obtained from 100 repeated holdout validations. In the holdout validation, 10,000 MOFs were randomly selected as the training dataset to build the LSTM model and the prediction values of the remaining MOFs were plotted in the figure. This procedure was repeated 100

times. If a data point is on the diagonal line, the prediction and actual values are the same.

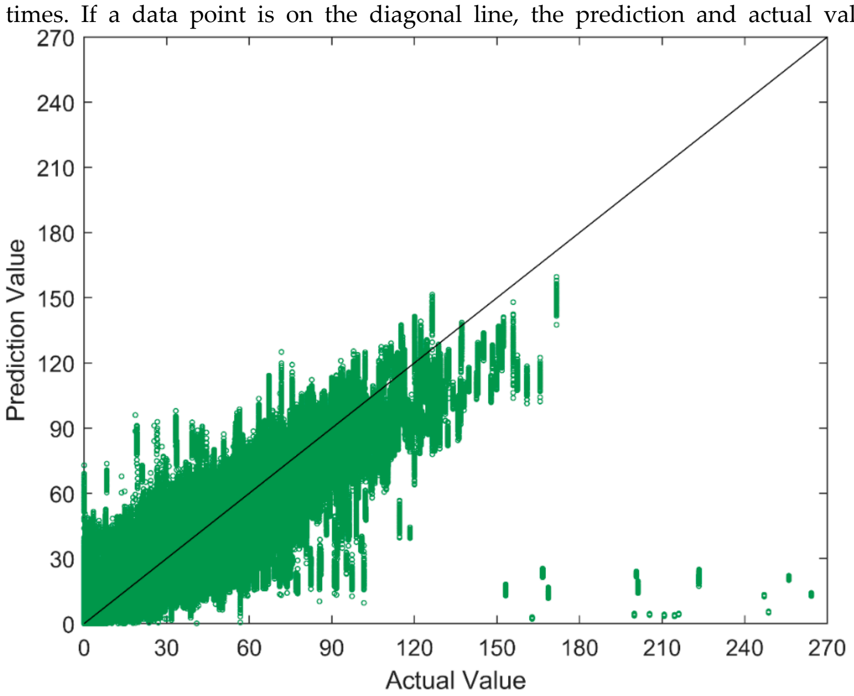

Figure S20. Performance of LSTM model using 26 descriptors in 100 repeated holdout validations for methane adsorption at 1 bar. The x-axis gives the actual values of volumetric-based methane adsorption in MOFs in units of cm^3(STP)/cm^3 at 1 bar and the y-axis gives the predicted values from the MLP model. The prediction values were obtained from 100 repeated holdout validations. In the holdout validation, 10,000 MOFs were randomly selected as the training dataset to build the MLP model and the prediction values of the remaining MOFs were plotted in the figure. This procedure was repeated 100 times. If a data point is on the diagonal line, the prediction and actual values are the same.

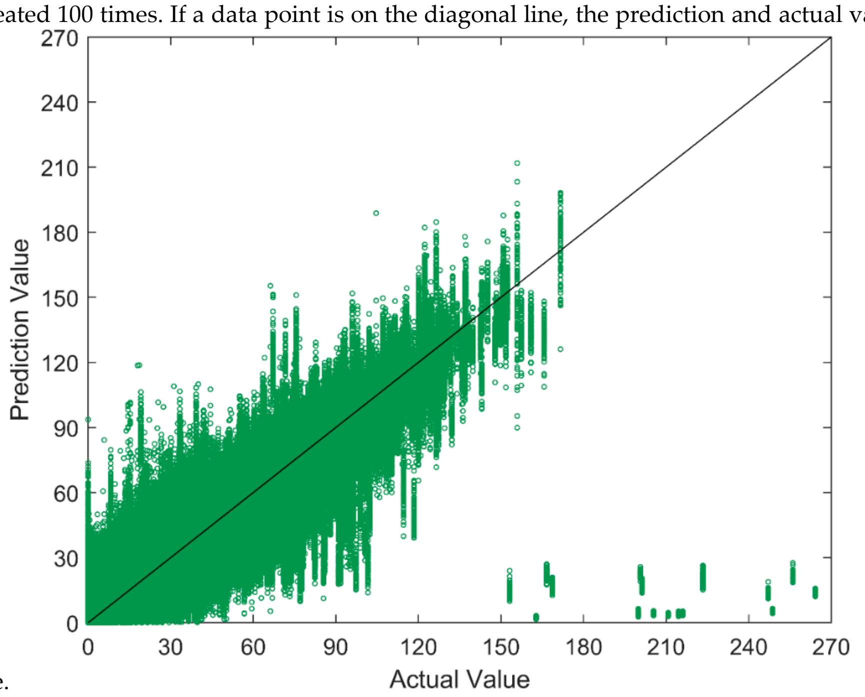

Figure S21. Performance of MLP model using 26 descriptors in 100 repeated holdout validations for methane adsorption at 1 bar. The x-axis gives the actual values of volumetric-based methane adsorption in MOFs in units of cm^3(STP)/cm^3 at 1 bar and the y-axis gives the predicted values from the MLP model. The prediction values were obtained from 100 repeated holdout validations. In the holdout validation, 10,000 MOFs were randomly selected as the training dataset to build the MLP model and the prediction values of the remaining MOFs were plotted in the figure. This procedure

was repeated 100 times. If a data point is on the diagonal line, the prediction and actual values are

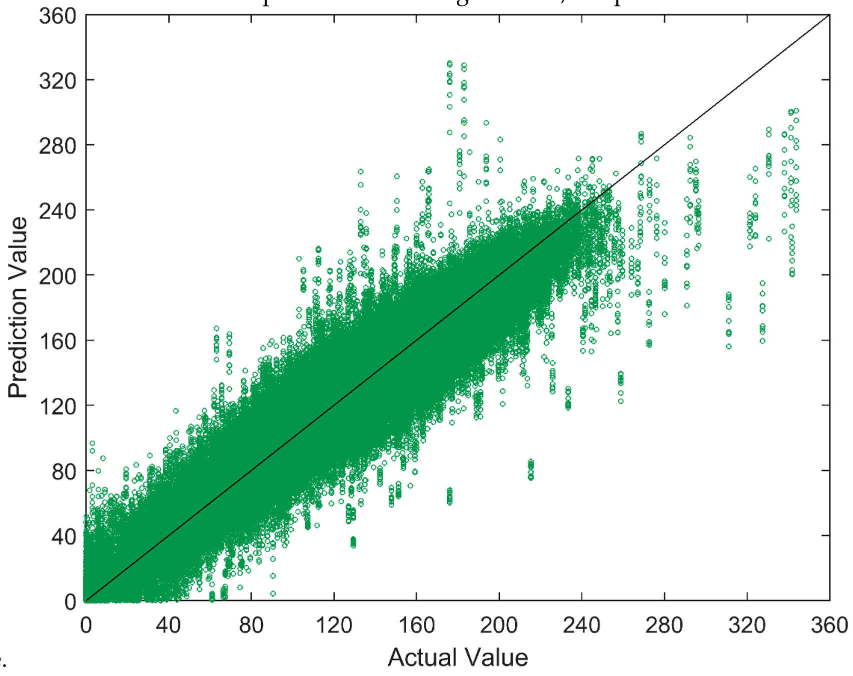

the same.

Figure S22. Performance of MLP model in 10-fold cross validations for carbon dioxide adsorption at 2.5 bar. The x-axis gives the actual values of volumetric-based carbon dioxide adsorption capacities in MOFs in units of cm^3(STP)/cm^3 at 2.5 bar and the y-axis gives the predicted values from the MLP model. The prediction values were obtained from 10 repeated 10-fold cross validations. If a data point is on the diagonal line, the prediction and actual values are the same.

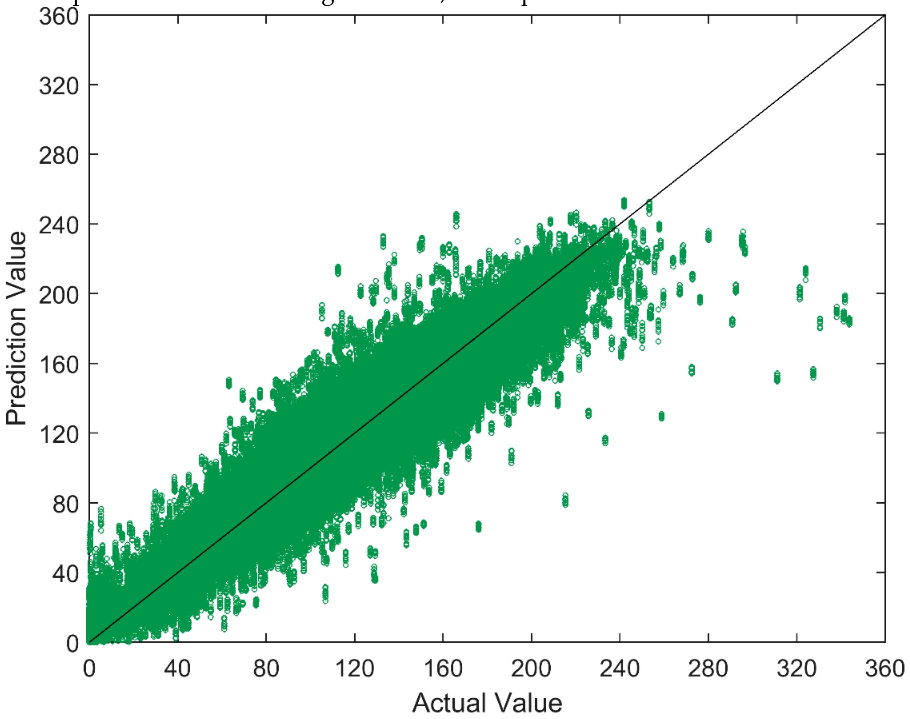

Figure S23. Performance of LSTM model in 10-fold cross validations for carbon dioxide adsorption at 2.5 bar. The x-axis gives the actual values of volumetric-based carbon dioxide adsorption capacities in MOFs in units of cm^3(STP)/cm^3 at 2.5 bar and the y-axis gives the predicted values from the LSTM model. The prediction values were obtained from 10 repeated 10-fold cross validations. If a data point is on the diagonal line, the prediction and actual values are the same.

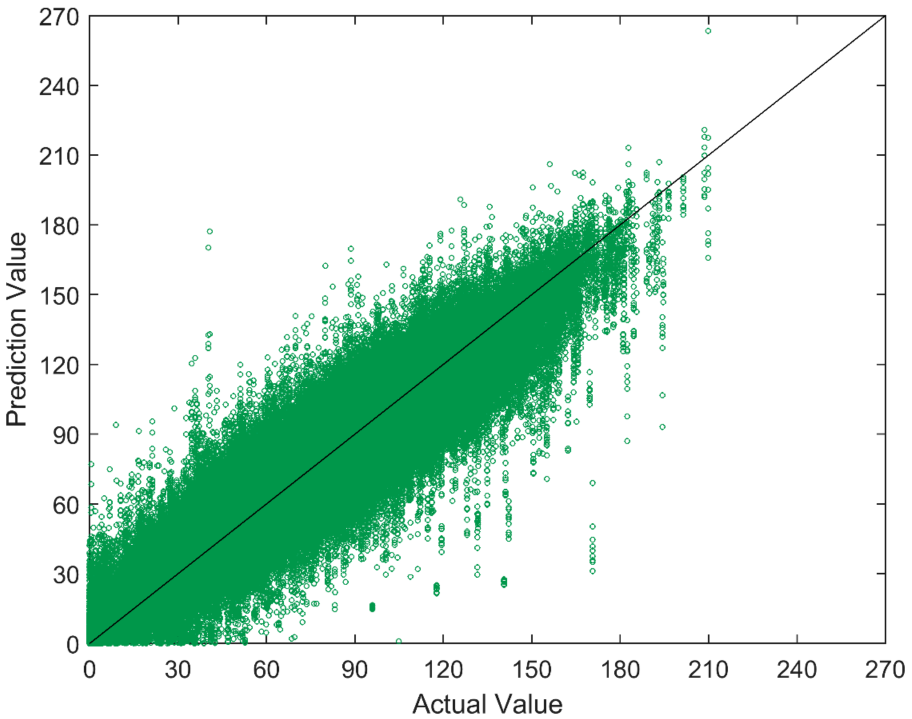

Figure S24. Performance of MLP model in 10-fold cross validations for carbon dioxide adsorption at 0.5 bar. The x-axis gives the actual values of volumetric-based carbon dioxide adsorption capacities in MOFs in units of cm^3(STP)/cm^3 at 0.5 bar and the y-axis gives the predicted values from the MLP model. The prediction values were obtained from 10 repeated 10-fold cross validations. If a data point is on the diagonal line, the prediction and actual values are the same.

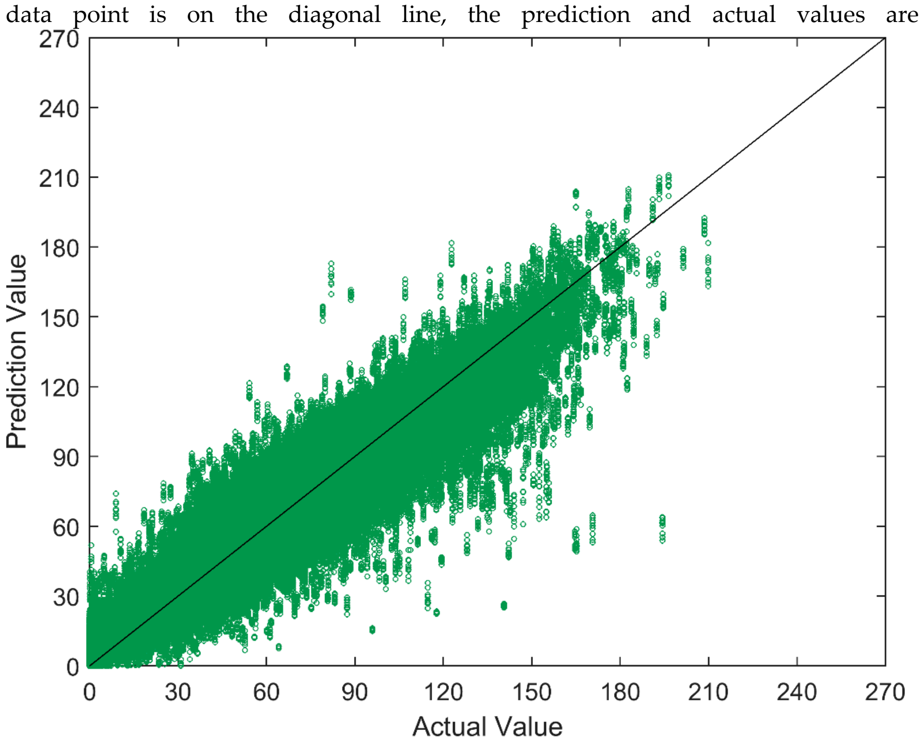

Figure S25. Performance of LSTM model in 10-fold cross validations for carbon dioxide adsorption at 0.5 bar. The x-axis gives the actual values of volumetric-based carbon dioxide adsorption capacities in MOFs in units of cm^3(STP)/cm^3 at 0.5 bar and the y-axis gives the predicted values from the LSTM model. The prediction values were obtained from 10 repeated 10-fold cross validations. If a

data point is on the diagonal line, the prediction and actual values are the same.

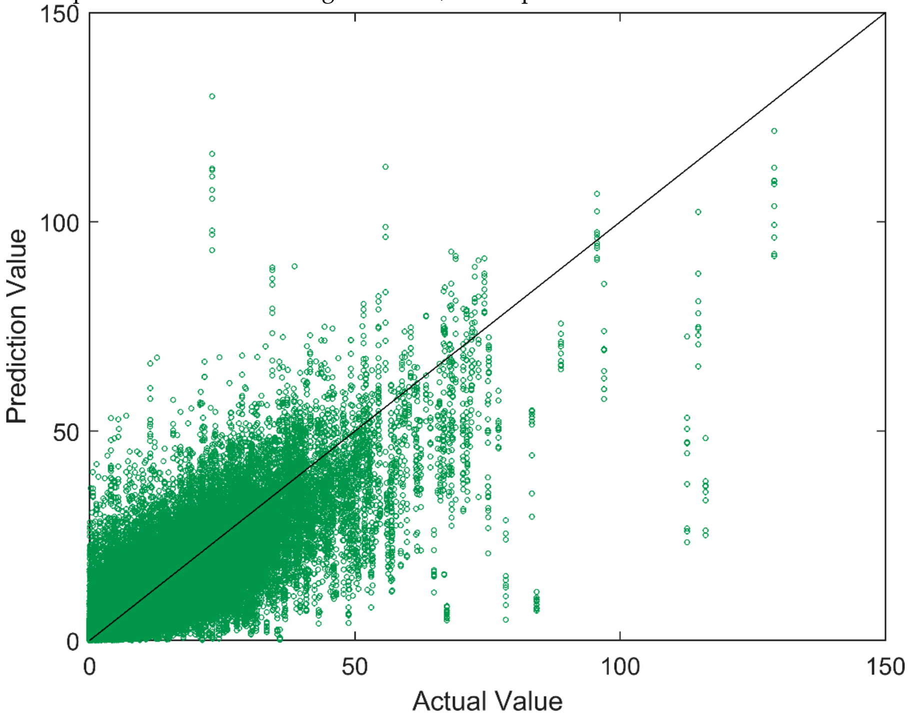

Figure S26. Performance of MLP model in 10-fold cross validations for carbon dioxide adsorption at 0.05 bar. The x-axis gives the actual values of volumetric-based carbon dioxide adsorption capacities in MOFs in units of cm^3(STP)/cm^3 at 0.05 bar and the y-axis gives the predicted values from the MLP model. The prediction values were obtained from 10 repeated 10-fold cross validations. If a data point is on the diagonal line, the prediction and actual values are the same.

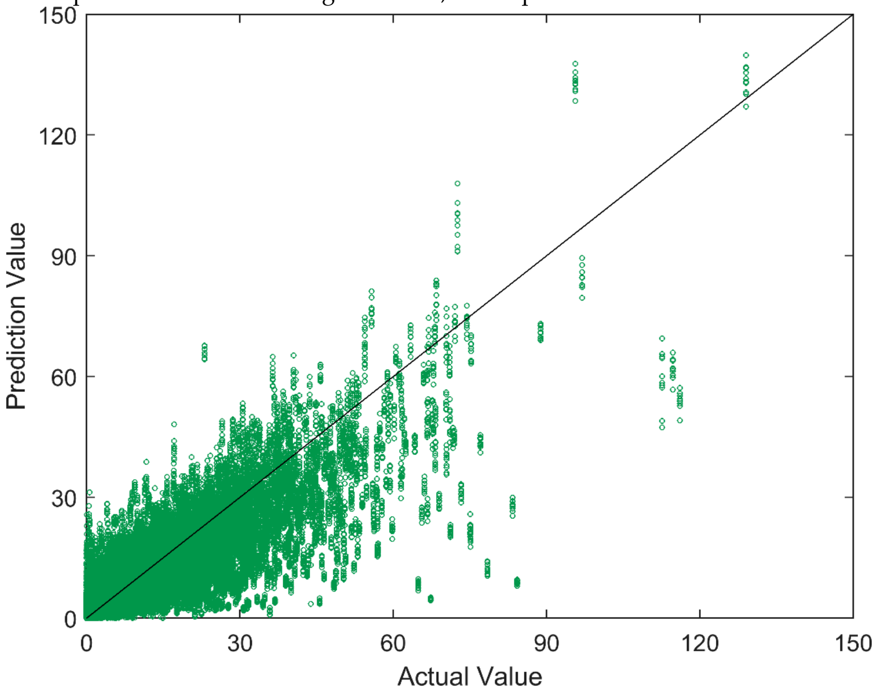

Figure S27. Performance of LSTM model in 10-fold cross validations for carbon dioxide adsorption at 0.05 bar. The x-axis gives the actual values of volumetric-based carbon dioxide adsorption capacities in MOFs in units of cm^3(STP)/cm^3 at 0.05 bar and the y-axis gives the predicted values from the LSTM model. The prediction values were obtained from 10 repeated 10-fold cross validations. If a

data point is on the diagonal line, the prediction and actual values are the same.

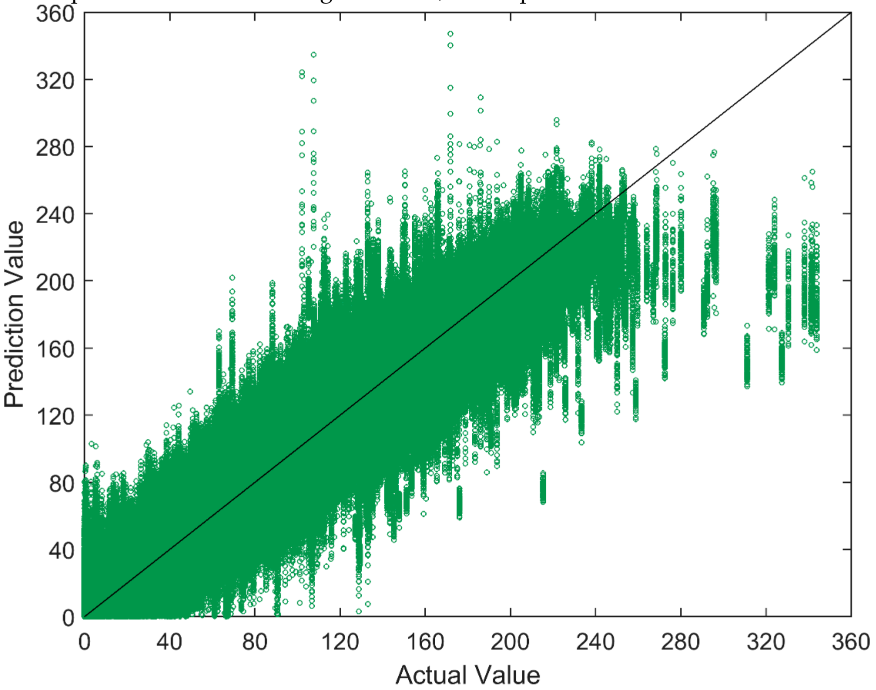

Figure S28. Performance of MLP model in 100 repeated holdout validations for carbon dioxide adsorption at 2.5 bar. The x-axis gives the actual values of volumetric-based carbon dioxide adsorption capacities in MOFs in units of cm^3(STP)/cm^3 at 2.5 bar and the y-axis gives the predicted values from the MLP model. The prediction values were obtained from 100 repeated holdout validations. In the holdout validation, 10,000 MOFs were randomly selected as the training dataset to build the MLP model and the prediction values of the remaining MOFs were plotted in the figure. This procedure was repeated 100 times. If a data point is on the diagonal line, the prediction and actual values are the same.

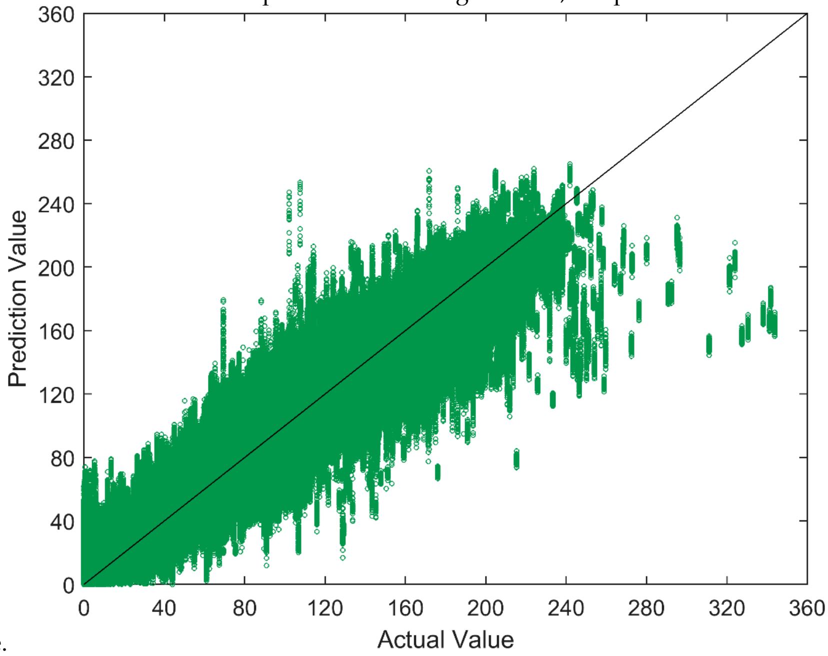

Figure S29. Performance of LSTM model in 100 repeated holdout validations for carbon dioxide adsorption at 2.5 bar. The x-axis gives the actual values of volumetric-based carbon dioxide adsorption capacities in MOFs in units of cm^3(STP)/cm^3 at 2.5 bar and the y-axis gives the predicted values from the LSTM model. The prediction values were obtained from 100 repeated holdout validations. In the holdout validation, 10,000 MOFs were randomly selected as the training dataset to build the LSTM model and the prediction values of the remaining MOFs were plotted in the figure. This

procedure was repeated 100 times. If a data point is on the diagonal line, the prediction and actual

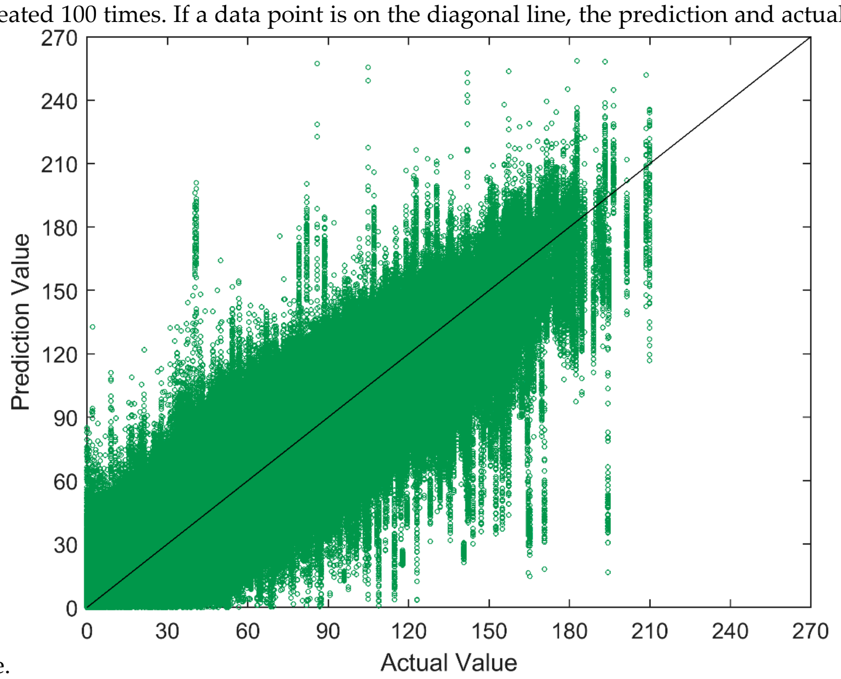

values are the same.

Figure S30. Performance of MLP model in 100 repeated holdout validations for carbon dioxide adsorption at 0.5 bar. The x-axis gives the actual values of volumetric-based carbon dioxide adsorption capacities in MOFs in units of cm^3(STP)/cm^3 at 0.5 bar and the y-axis gives the predicted values from the MLP model. The prediction values were obtained from 100 repeated holdout validations. In the holdout validation, 10,000 MOFs were randomly selected as the training dataset to build the MLP model and the prediction values of the remaining MOFs were plotted in the figure. This procedure was repeated 100 times. If a data point is on the diagonal line, the prediction and actual values are

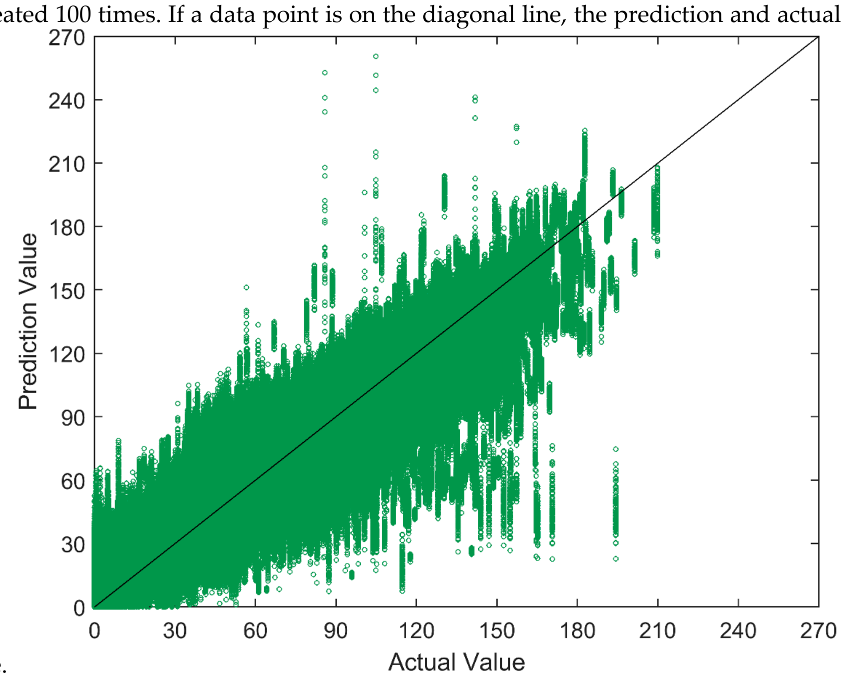

the same.

Figure S31. Performance of LSTM model in 100 repeated holdout validations for carbon dioxide adsorption at 0.5 bar. The x-axis gives the actual values of volumetric-based carbon dioxide adsorption capacities in MOFs in units of cm^3(STP)/cm^3 at 0.5 bar and the y-axis gives the predicted values from the LSTM model. The prediction values were obtained from 100 repeated holdout validations. In the holdout validation, 10,000 MOFs were randomly selected as the training dataset to build the LSTM model and the prediction values of the remaining MOFs were plotted in the figure. This

procedure was repeated 100 times. If a data point is on the diagonal line, the prediction and actual values are the same.

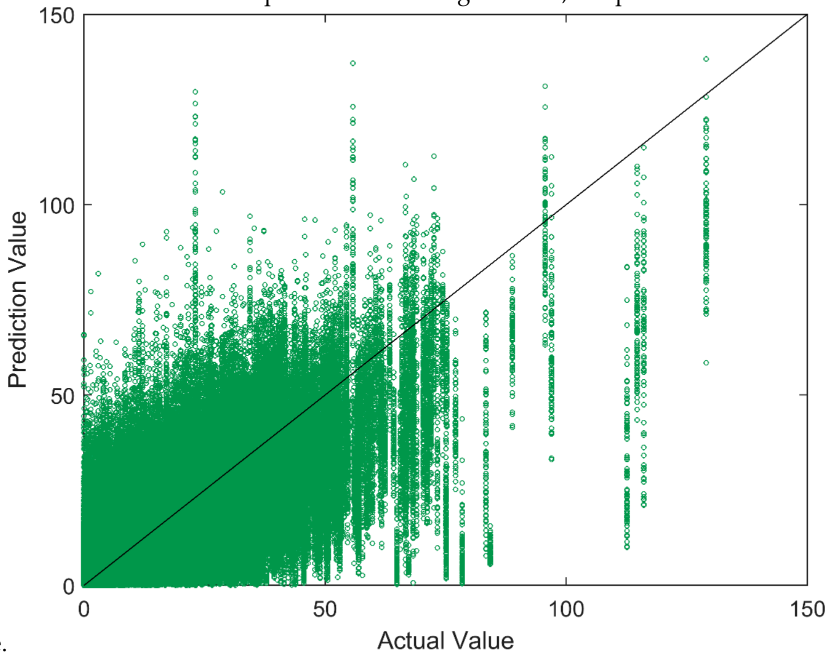

Figure S32. Performance of MLP model in 100 repeated holdout validations for carbon dioxide adsorption at 0.05 bar. The x-axis gives the actual values of volumetric-based carbon dioxide adsorption capacities in MOFs in units of cm^3(STP)/cm^3 at 0.05 bar and the y-axis gives the predicted values from the MLP model. The prediction values were obtained from 100 repeated holdout validations. In the holdout validation, 10,000 MOFs were randomly selected as the training dataset to build the MLP model and the prediction values of the remaining MOFs were plotted in the figure. This procedure was repeated 100 times. If a data point is on the diagonal line, the prediction and actual values are the same.

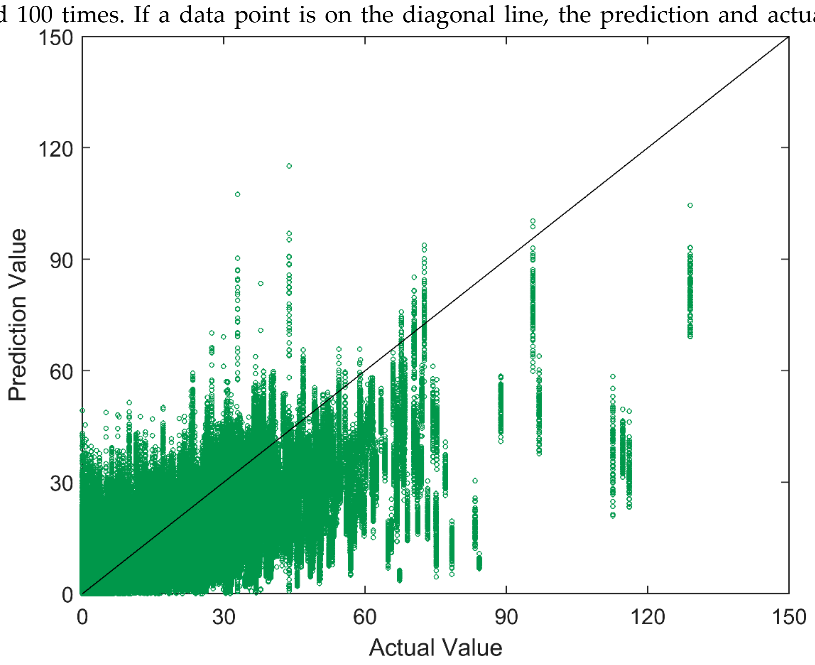

Figure S33. Performance of LSTM model in 100 repeated holdout validations for carbon dioxide adsorption at 0.05 bar. The x-axis gives the actual values of volumetric-based carbon dioxide adsorption capacities in MOFs in units of cm^3(STP)/cm^3 at 0.05 bar and the y-axis gives the predicted values from the LSTM model. The prediction values were obtained from 100 repeated holdout validations. In the holdout validation, 10,000 MOFs were randomly selected as the training dataset to build the LSTM model and the prediction values of the remaining MOFs were plotted in the figure. This procedure was repeated 100 times. If a data point is on the diagonal line, the prediction and actual values are the same.

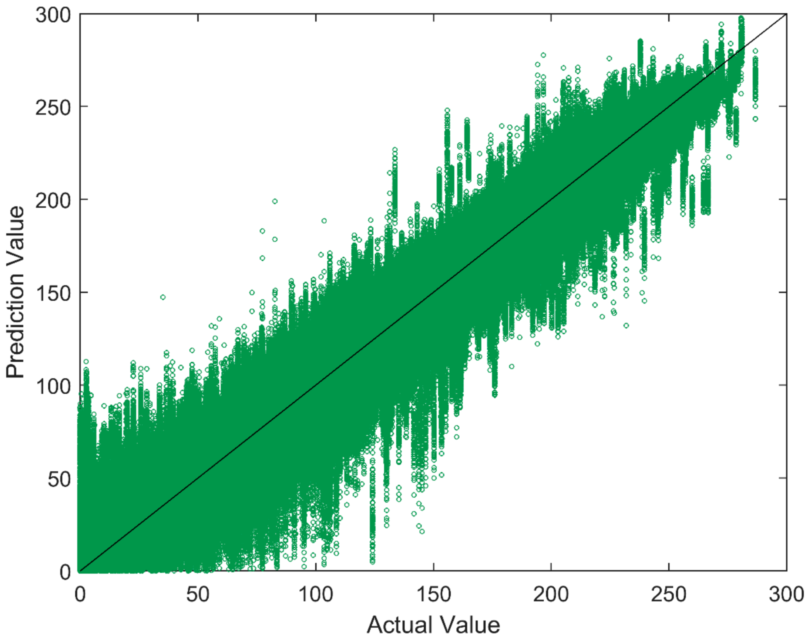

Figure S34. Performance of MLP model in 100 repeated holdout validations for methane adsorption in the mixture of MOFs and COFs at 65 bar. The x-axis gives the actual values of methane adsorption in the mixture of MOFs and COFs at 65 bar and the y-axis gives the predicted values from the MLP model. The values are volumetric-based adsorption capacities in units of cm^3(STP)/cm^3. The prediction values were obtained from 100 repeated holdout validations. In the holdout validation, 5000 COFs and 5000 MOFs were randomly selected as the training dataset to build the MLP models and the prediction values of the remaining COFs and MOFs were plotted in the figure. This procedure was repeated 100 times. If a data point is on the diagonal line, the prediction and actual values are the same.

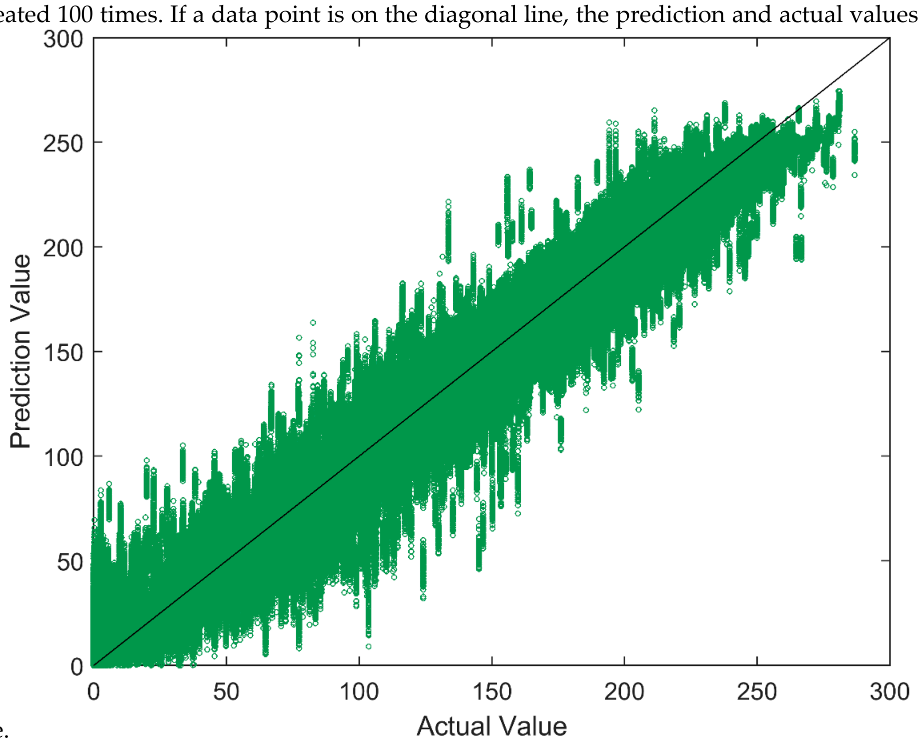

Figure S35. Performance of LSTM model in 100 repeated holdout validations for methane adsorption in the mixture of MOFs and COFs at 65 bar. The x-axis gives the actual values of methane adsorption in the mixture of MOFs and COFs at 65 bar and the y-axis gives the predicted values from the LSTM model. The values are volumetric-based adsorption capacities in units of cm^3(STP)/cm^3. The prediction values were obtained from 100 repeated holdout validations. In the holdout validation, 5000 COFs and 5000 MOFs were randomly selected as the training dataset to build the LSTM models and the prediction values of the remaining COFs and MOFs were plotted

in the figure. This procedure was repeated 100 times. If a data point is on the diagonal line, the prediction and actual values are the same.
